# PPARγ Negatively Regulates T Cell Activation to Prevent Follicular Helper T Cells and Germinal Center Formation

**DOI:** 10.1371/journal.pone.0099127

**Published:** 2014-06-12

**Authors:** Hong-Jai Park, Do-Hyun Kim, Jin-Young Choi, Won-Ju Kim, Ji Yun Kim, Alireza G. Senejani, Soo Seok Hwang, Lark Kyun Kim, Zuzana Tobiasova, Gap Ryol Lee, Joseph Craft, Alfred L. M. Bothwell, Je-Min Choi

**Affiliations:** 1 Department of Life Science, Research Institute for Natural Sciences, Hanyang University, Seoul, Korea; 2 Hanyang Biomedical Research Institute, Seoul, Korea; 3 Department of Internal Medicine (Rheumatology), Yale University School of Medicine, New Haven, Connecticut, United States of America; 4 Department of Therapeutic Radiology and Genetics, Yale University School of Medicine, New Haven, Connecticut, United States of America; 5 Department of Life Science, Sogang University, Seoul, Korea; 6 Department of Immunobiology, Yale University School of Medicine, New Haven, Connecticut, United States of America; The University of Texas Medical School at Houston, United States of America

## Abstract

Peroxisome proliferator-activated receptor gamma (PPARγ) is a transcription factor that regulates lipid and glucose metabolism. Although studies of PPARγ ligands have demonstrated its regulatory functions in inflammation and adaptive immunity, its intrinsic role in T cells and autoimmunity has yet to be fully elucidated. Here we used CD4-PPARγ^KO^ mice to investigate PPARγ-deficient T cells, which were hyper-reactive to produce higher levels of cytokines and exhibited greater proliferation than wild type T cells with increased ERK and AKT phosphorylation. Diminished expression of IκBα, Sirt1, and Foxo1, which are inhibitors of NF-κB, was observed in PPARγ-deficient T cells that were prone to produce all the signature cytokines under Th1, Th2, Th17, and Th9 skewing condition. Interestingly, 1-year-old CD4-PPARγ^KO^ mice spontaneously developed moderate autoimmune phenotype by increased activated T cells, follicular helper T cells (T_FH_ cells) and germinal center B cells with glomerular inflammation and enhanced autoantibody production. Sheep red blood cell immunization more induced T_FH_ cells and germinal centers in CD4-PPARγ^KO^ mice and the T cells showed increased of Bcl-6 and IL-21 expression suggesting its regulatory role in germinal center reaction. Collectively, these results suggest that PPARγ has a regulatory role for T_FH_ cells and germinal center reaction to prevent autoimmunity.

## Introduction

Nuclear receptors constitute a superfamily of ligand-dependent transcription factors that regulate diverse aspects of metabolism and homeostasis. This family is further subdivided into three subclasses based on their DNA-binding and ligand-binding properties, including classical steroid hormone receptors, adopted orphan receptors, and orphan receptors [1]–[4]. Peroxisome Proliferator-activated Receptors (PPAR) are emerging adopted orphan nuclear receptors that regulate lipid and glucose metabolism, as well as cell growth, differentiation, apoptosis, and immunity, in a wide variety of cells. The PPAR subfamily consists of three subtypes including PPARα, PPARβ/δ, and PPARγ. Each member binds multiple ligands, regulates target genes, and plays different biological roles. The ligands for PPARγ have been used for therapeutic applications to treat atherosclerosis, obesity-induced insulin resistance, and inflammatory bowel disease [5]–[7]. For example, synthetic PPARγ agonists called thiazolidinediones (TZD) improved insulin sensitivity in metabolic syndrome [5], [8]. The PPARγ agonist 15-deoxy-△^12,14^-prostaglandin J_2_ (15-PGJ_2_) has been shown to downregulate T cell proliferation and IL-2 production [9]. Another PPARγ agonist, ciglitazone, has been shown to increase regulatory T (Treg) cell differentiation from naïve T cells [10], while pioglitazone has been demonstrated to ameliorate EAE severity through selective inhibition of Th17 differentiation [11] and inhibit human alloresponses in a humanized model of graft arteriosclerosis [12]. More recently, PPARγ has been shown to regulate Treg accumulation in visceral adipose tissue, and pioglitazone treatment was required for complete recovery from insulin sensitivity in obese mice [13].

Autoimmune disease occurs when the immune system recognizes self-molecules and responds by inducing autoantibody production, germinal center (GC) formation, and chronic inflammation [14]–[16]. Follicular helper T (T_FH_) cells are involved in the antibody response by providing selection signals to GC B cells and promoting plasma and memory cell differentiation [17]. Bcl-6 mediates the development of T_FH_ cells, which are characterized by expression of the chemokine receptor CXCR5, ICOS, and PD-1 [18]–[21]. Recent studies have suggested T_FH_ cells as a potential therapeutic target for treating autoimmune diseases [15], [22], [23].

Although previous studies focused on PPARγ agonists revealed its regulatory function in T cells, the mechanism and intrinsic roles of this nuclear receptor in T_FH_ cells and autoimmunity is largely uncharacterized. Therefore, we investigated the role of PPARγ in T cells using CD4 T cell-specific PPARγ knockout mice. Interestingly, we found that PPARγ-deficient T cells exhibit enhanced proliferation following T cell receptor (TCR) stimulation and increased cytokine production under Th1, Th2, Th17, and Th9 differentiation conditions. Interestingly moderate autoimmune phenotypes developed spontaneously in 1-year-old CD4-PPARγ^KO^ mice with increased activated T cells, T_FH_ cells, and GCs. Furthermore, PPARγ regulated T cell activation by modulating the stability of IκBα, Foxo1, and Sirt1, which are negative regulators of NF-κB and T_FH_ cells. These results suggest the important regulatory roles of PPARγ in T_FH_ cells and GC formation in the prevention of autoimmunity.

## Results

### PPARγ is highly expressed in CD4 T cells upon TCR stimulation

**Figure 1:**
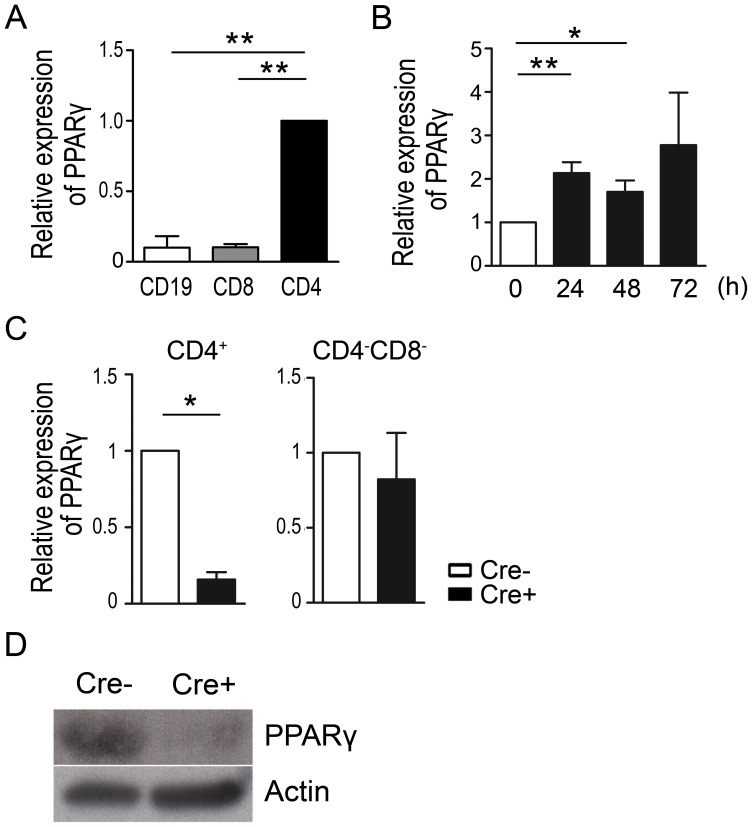
PPARγ expression in T cells and generation of CD4-PPARγ^KO^ mice. (A) Expression of PPARγ in FACS-purified CD4^+^, CD8^+^, and CD19^+^ cells from female wild type C57BL/6J mice was analyzed by quantitative real-time PCR and normalized to β-actin. Values represent the mean ± SEM, n = 4. **P<0.01 when CD19 and CD8 were compared. (B) MACS-purified naïve (CD4^+^CD25^−^CD62L^high^CD44^low^) T cells from female wild type C57BL/6J mice were stimulated with plate-bound anti-CD3 and soluble anti-CD28 antibodies for the indicated times. PPARγ expression was determined by quantitative real-time PCR. Relative PPARγ expression level was calculated as the fold-change relative to 0 h. Values represent the mean ± SEM, n = 4. *P<0.05, **P<0.01 when 0 h, 24 h, and 48 h were compared. (C) FACS-sorted cells from female littermate control (Cre-) and CD4-PPARγ^KO^ (Cre+) mice were harvested and PPARγ expression was determined by quantitative real-time PCR. Values represent the mean ± SEM, n = 3. *P<0.05. (D) Cre-mediated PPARγ deletion in cell lysate prepared from CD4^+^ T cells was confirmed by western blot analysis.

To determine its expression in various lymphocyte subsets, the level of PPARγ mRNA was examined in FACS-purified CD4^+^ T, CD8^+^ T, and CD19^+^ B cells by quantitative real-time PCR analysis. PPARγ expression was significantly higher in CD4^+^ T cells compared to CD8^+^ T cells and CD19^+^ B cells (Figure 1A), suggesting that this nuclear receptor may play important roles in CD4^+^ T cells. In addition, PPARγ mRNA expression was significantly increased following TCR stimulation (Figure 1B), further implying its regulatory role in controlling activated CD4^+^ T cell functions.

Next, we generated mice in which PPARγ was specifically deleted in CD4^+^ T cells by breeding PPARγ^fl/fl^ with CD4-Cre transgenic mice (Figure 1C&D). PPARγ-deficiency did not affect the frequency of CD4^+^, CD8^+^, and Foxp3^+^ natural regulatory T cell (nTreg) in the thymus of 6- to 8-week-old mice (Figure S1A-D), the proportion of lymphocyte population including B cells, NK cells, and naïve or memory T cells in the spleen (Figure S1E-L). Altogether these data suggest that PPARγ deletion in CD4^+^ T cells does not affect thymic and splenic cell populations that PPARγ is dispensable for T cell development.

### PPARγ-deficient T cells are hyper-reactive to TCR stimulation

**Figure 2:**
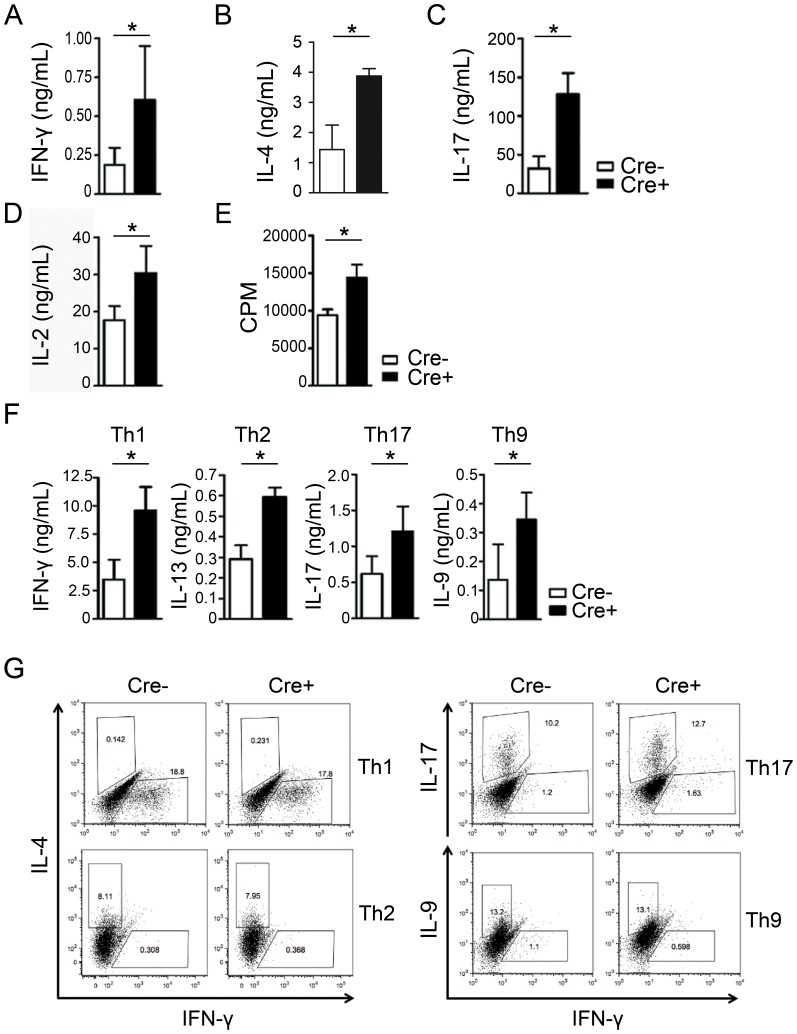
PPARγ deficiency induces hyper-reactivity in T cells. CD4^+^CD25^−^ T cells from the spleens of 6- to 8-week-old female littermate control (Cre−) and CD4-PPARγ^KO^ (Cre+) mice were stimulated for 24 h with plate-bound anti-CD3 and soluble anti-CD28 antibodies. (A) IFN-γ, (B) IL-4, (C) IL-17, and (D) IL-2 levels in culture supernatants were measured by ELISA. (E) Proliferation of 3-day-anti-CD3/CD28 stimulated cells was measured by H^3^-thymidine incorporation. Values represent the mean ± SEM of counts per minute (CPM) in triplicate wells. CD4^+^25^−^ T cells were differentiated under Th1, Th2, Th17, and Th9 differentiation conditions. (F) After 5 days, the culture supernatants were collected and cytokine levels (IFN-γ, IL-13, IL-17, and IL-9) were measured by ELISA. Values represent the mean ± SEM, n = 4–6. *P<0.05. (G) Flow cytometric analysis for lineage-specific cytokines (IFN-γ, IL-4, IL-17, and IL-9) of Th1, Th2, Th17 and Th9 were determined by intracellular staining. Representative data were shown from five independent experiments.

Although PPARγ is dispensable for lymphocyte development, increased PPARγ expression following TCR stimulation led us to examine its function during T cell activation. To address this, we examined cytokine production from purified CD4^+^CD25^−^ T cells stimulated with anti-CD3 and anti-CD28 antibodies. Various cytokines including IFN-γ, IL-4, IL-17, and IL-2 were increased in PPARγ-deficient T cells compared to littermate control T cells, (Figure 2A-D) suggesting that these cells are hyper-reactive to TCR stimulation. In addition, PPARγ-deficient T cells proliferated significantly more than control CD4^+^ T cells (Figure 2E), suggesting that PPARγ plays a role as a negative regulator of T cell activation and proliferation.

Next, we investigated cytokine production after CD4^+^CD25^−^ T cells were differentiated into various helper T cell subsets including Th1, Th2, Th17, and Th9. Interestingly, production of all the signature cytokines secreted by each helper T cell subset, namely IFN-γ, IL-13, IL-17, and IL-9, respectively, were significantly increased in PPARγ-deficient T cells (Figure 2F) without affecting differentiation efficiency (Figure 2G). These results suggest that PPARγ has a negative regulatory role for T cell activation and proliferation.

### PPARγ maintains the stability of IκBα, Foxo1, and Sirt1 to regulate TCR signaling

**Figure 3:**
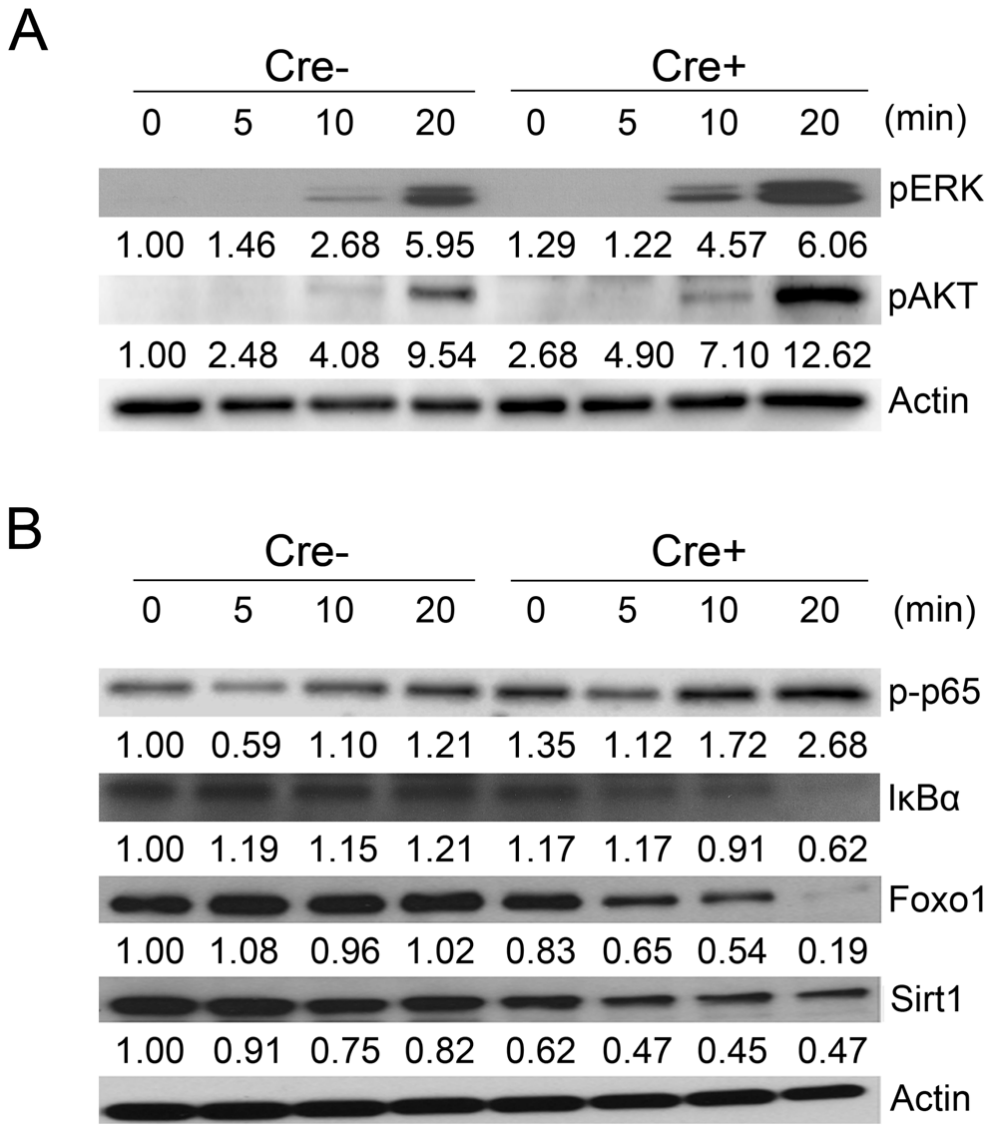
PPARγ deficiency in T cells reduces the expression of molecules that inhibit NF-κB. MACS-purified CD4^+^CD25^−^CD62L^high^ naive T cells from the spleens of 6- to 8-week-old female littermate control (Cre−) and CD4-PPARγ^KO^ (Cre+) mice were stimulated with anti-CD3 and anti-CD28 Dynabeads for the indicated times. (A) Levels of ERK and AKT phosphorylation and (B) Levels of p65 phosphorylation, IκBα, Foxo1, and Sirt1 were assessed by western blot analysis. The arbitrary unit represents band intensity normalized to β-actin and the relative value to WT 0 min is indicated. Representative data were shown from at least two independent experiments.

A previous study reported that PPARγ inhibits NF-κB in macrophages [24]. However, the molecular basis of how PPARγ regulates T cell activation has not been fully elucidated. To determine the mechanism, we assessed ERK and AKT phosphorylation in splenic naïve CD4^+^ T cells (CD4^+^CD25^−^CD62L^high^) stimulated with anti-CD3/CD28-coated beads. Following TCR stimulation for 20 min, the PPARγ-deficient CD4^+^ T cells showed increased ERK and AKT phosphorylation compared to littermate control T cells (Figure 3A), suggesting that PPARγ inhibits events of TCR downstream signaling pathway.

We performed microarray analysis to identify possible target genes (e.g., Foxo1) regulated by PPARγ (data not shown). Interestingly, TCR stimulation of PPARγ-deficient CD4^+^ T cells resulted in reduced expression of IκBα, Foxo1, and Sirt1 (Figure 3B), which have been recently identified as negative regulators of T cell activation [25], [26]. In addition, phosphorylated p65 level was increased in PPARγ-deficient CD4^+^ T cells suggesting that PPARγ contributes to the stability of IκBα, Foxo1, and Sirt1 to regulate NF-κB in activated T cells.

### PPARγ deficiency in T cells spontaneously enhances activated T cells in vivo

**Figure 4:**
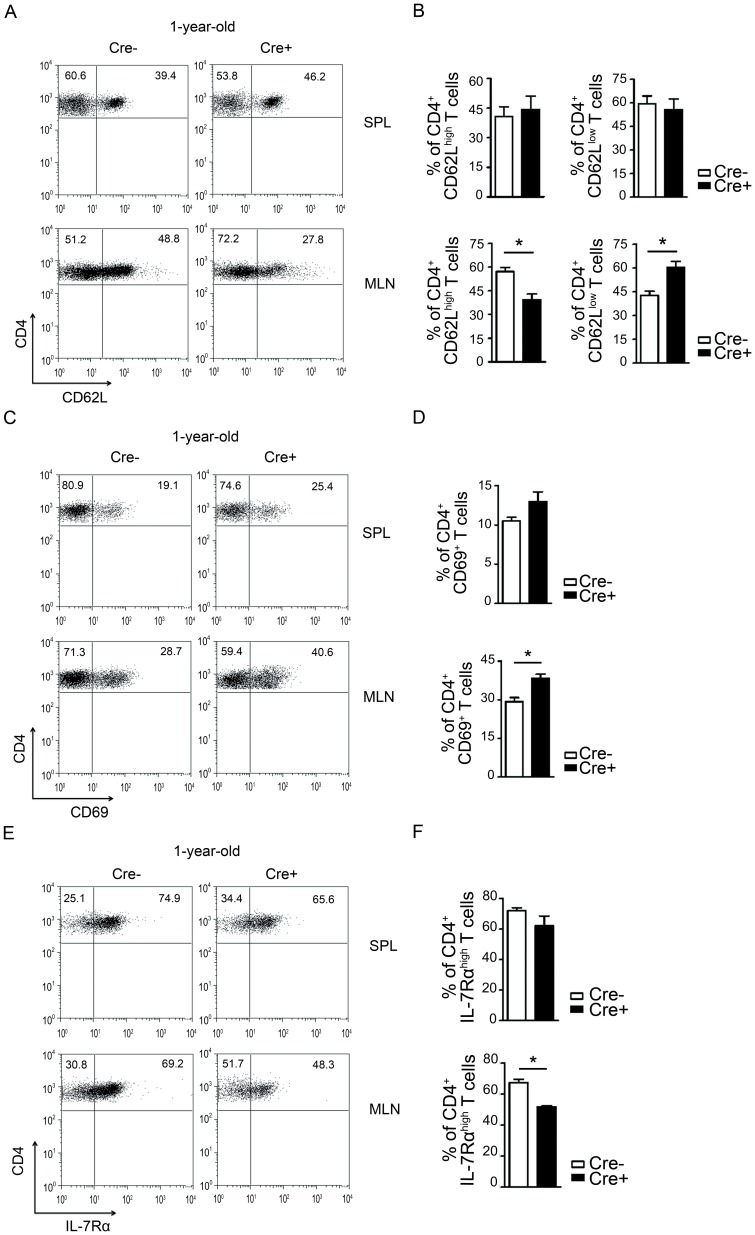
Increase of CD4^+^CD62L^low^, CD4^+^CD69^+^ and CD4^+^IL-7Rα^high^ cells in 1-year-old CD4-PPARγ^KO^ mice. Lymphocytes from spleen (SPL) and mesenteric lymph nodes (MLN) were isolated from 1-year-old control (Cre−) and CD4-PPARγ^KO^ (Cre+) mice and then stained with anti-CD4, anti-CD62L, anti-CD69 and anti-IL-7Rα antibodies for flow cytometric analysis. (A, B) CD4^+^CD62L^low^ cells, (C, D) CD4^+^CD69^+^ cells, and (E, F) CD4^+^IL-7Rα^high^ cells were analyzed. Values represent the mean ± SEM, n = 5-6. *P<0.05.

The TCR-stimulated hyper-reactivity observed in PPARγ-deficient T cells from 6- to 8-week-old CD4-PPARγ^KO^ mice prompted us to investigate spontaneous changes in the T cell phenotype of 1-year-old CD4-PPARγ^KO^ mice. CD62L is a cell adhesion molecule, which is highly expressed on naïve T lymphocytes. The frequency of CD4^+^CD62L^low^ cells was increased significantly in the mesenteric lymph nodes (MLN) of CD4-PPARγ^KO^ mice compared to age-matched control mice (Figure 4A&B). The proportion of CD4^+^CD62L^low^ cells of CD4-PPARγ^KO^ mice was augmented compared to littermate control. These results are supported by an increase of the early activation marker CD69 expressing CD4^+^ T cells (Figure 4C&D). Additionally, we investigated the expression of IL-7Rα, which has been shown to be decreased in activated T cells [27]. PPARγ deficiency in T cells from 1-year-old CD4-PPARγ^KO^ mice showed significantly reduced proportion of CD4^+^IL-7Rα^high^ cells in the MLN (Figure 4E&F). Collectively, these data demonstrate that PPARγ deficiency in T cells contributes to their hyper-reactivity, which induces spontaneous T cell activation *in vivo*.

### PPARγ-deficient T cells in mice develop a spontaneous autoimmune phenotype

**Figure 5:**
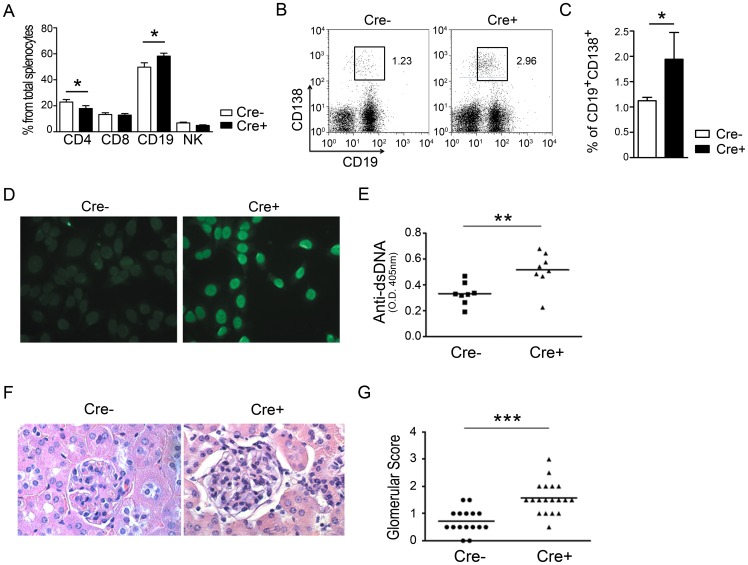
PPARγ deficiency in T cells results in spontaneous autoimmune phenotypes. (A) Percentages of CD4, CD8, CD19, and NK in the spleen of 6-month-old control (Cre−) and CD4-PPARγ^KO^ (Cre+) mice. Values represent the mean ± SEM, n = 10. *P<0.05. (B, C) Comparison of the frequency of CD19^+^CD138^+^ plasma cells in female control (Cre−) and CD4-PPARγ^KO^ (Cre+) splenocytes. Values represent the mean ± SEM, n = 6. *P<0.05. (D) The level of anti-nuclear antibody in the serum of 6-month-old mice was examined by immunofluorescence of pre-fixed NIH3T3 cells stained with diluted (1:100) sera followed by Alexa Fluor 488-conjugated anti-mouse Ig antibody. (E) Quantitation of anti-dsDNA antibody in serum of 1-year-old mice by ELISA, n = 8. (F) Hematoxylin and eosin staining of kidney from 1-year-old mice. (G) The severity of inflammation in 1-year-old mice, n = 16–19. *P<0.05; ** *P*<0.01; *** *P*<0.001.

Because the number of activated T cells was increased in 1-year-old CD4-PPARγ^KO^ mice, we next investigated autoimmune phenotype in these animals. Flow cytometric analysis of splenocytes from 6-month-old CD4-PPARγ^KO^ mice demonstrated that the proportion of CD19^+^ B cells was increased while CD4^+^ T cells were slightly decreased compared to littermate control mice (Figure 5A). Based on this observation, we hypothesized that the hyper-reactive PPARγ-deficient T cells promote greater B cell activation, thereby leading to autoantibody production. Our data reveal that the proportion of CD19^+^ and CD138^+^ plasma cells was increased in the spleen of CD4-PPARγ^KO^ mice (Figure 5B&C). Moreover, the level of anti-nuclear antibodies (ANA) in serum from 6-month-old mice (Figure 5D) and the level of anti-double-stranded DNA (dsDNA) antibodies in serum from 1-year-old mice (Figure 5E) were higher than age-matched control mice.

To further examine the autoimmune phenotype, inflammation in the kidneys was examined and scored by histologic analysis of hematoxylin and eosin-stained sections (Figure 5F&G). Kidney from 1-year-old CD4-PPARγ^KO^ mice exhibited an expanded double layer around the glomerulus with thicker tubules compared to age-matched control mice. These results suggest that PPARγ deficiency in T cells leads to autoantibody production and spontaneous autoimmune disease in aged mice.

### PPARγ-deficient T cells contribute to T_FH_ cells and GC development

**Figure 6:**
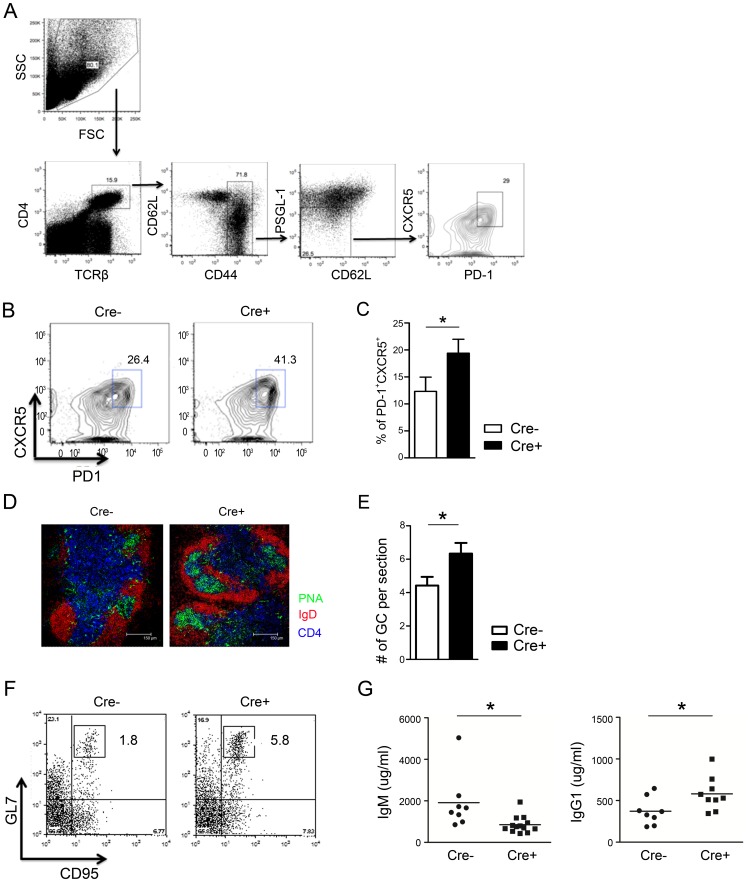
PPARγ deficiency in T cells spontaneously increases follicular helper T cell and germinal center formation. (A) Representative flow cytometric gating strategy for the identification of follicular helper T cells, which were identified as CD4^+^TCRβ^+^CD62L^low^CD44^high^PSGL-1^low^CXCR5^+^PD-1^+^ cells in the spleen of 1-year-old mice. (B, C) Flow cytometric analysis of T_FH_ cells from 1-year-old mice. Values represent the mean ± SEM, n = 10. *P<0.05. (D) Germinal centers of frozen 7-µm sections from spleen of 1-year-old mice were visualized by confocal microscopy. Slides were stained for PNA (green), IgD (red), and CD4 (blue) to detect germinal centers, B cells, and T cells, respectively. (E) The number of PNA^+^ germinal centers per spleen section was quantitated, n = 5. (F) The frequency of B220^+^GL7^+^CD95^+^ germinal center B cells from splenocytes and (G) amount of IgM and IgG1 from the sera of 1-year-old control (Cre−) and CD4-PPARγ^KO^ (Cre+) mice was analyzed. Values represent the mean ± SEM, n = 8–11. *P<0.05.

T_FH_ cells are crucial for providing help to B cells within the germinal center, where somatic hyper-mutation, class-switch recombination, and differentiation of memory B cells and long-lived plasma cells occur [28]–[31]. Due to increased B cell numbers, including plasma cells, in 6-month-old CD4-PPARγ^KO^ mice, we hypothesized that PPARγ deficiency in T cells lead to increased T_FH_ cells *in vivo*. Previous report has shown that PSGL-1 low population enriched more T_FH_ cells than PSGL-1 high [31] so that PSGL-1^low^ gating strategy was applied to analyze T_FH_ cells. Flow cytometric analysis revealed an increased proportion of CXCR5^+^PD-1^+^ T_FH_ cells in 1-year-old CD4-PPARγ^KO^ mice (Figure 6A - C). The number of GCs found in the spleen of these animals was also increased compared to age-matched control mice (Figure 6D&E). In addition, proportion of germinal center B cells (CD95^+^GL7^+^ from B220^+^ cells) were increased in the spleen with elevated amount of IgG1 and reduced IgM from the sera in 1-year-old CD4-PPARγ^KO^ mice (Figure 6F&G). These results suggest that the spontaneous autoimmune phenotypes observed were related to increased T_FH_ cells and enhanced GC reaction.

**Figure 7:**
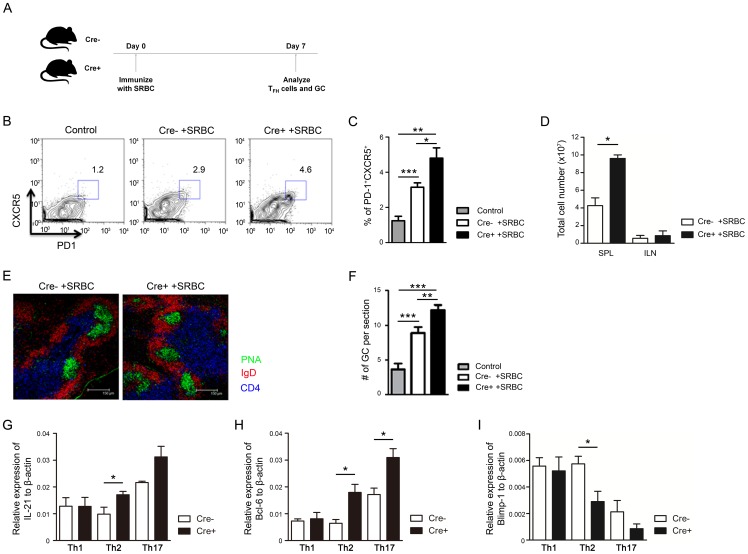
PPARγ deficiency in T cells increases follicular helper T cell and germinal center formation following SRBC immunization. (A) Experimental scheme for SRBC immunization to analyze T_FH_ cells and GC reaction. (B, C) Flow cytometric analysis of T_FH_ cells in the spleen and (D) total cell number of spleens and lymph nodes from 8-week-old control and SRBC-immunized mice. Values represent the mean ± SEM, n = 7–10. *P<0.05, **P<0.01, ***P<0.001. (E) Germinal centers of frozen 7-µm sections from spleen of 8-week-old control (non-immunized Cre-) and SRBC-immunized mice were visualized by confocal microscopy. Slides were stained for PNA (green), IgD (red), and CD4 (blue) to detect germinal centers, B cells, and T cells, respectively. (F) The number of PNA^+^ germinal centers per spleen section was quantitated n = 5–7. Expression level of (G) IL-21, (H) Bcl-6 and (I) Blimp-1 was assessed by real-time PCR and normalized to β-actin. Values represent the mean ± SEM, n = 3. **P<0.01, ***P<0.001.

To further confirm that PPARγ regulates induction of T_FH_ cells and GC reaction, 8-week-old mice were immunized with sheep red blood cells (SRBC) and the T_FH_ cells and GC number were analyzed on day 7 (Figure 7A). Following SRBC immunization, the proportion of CXCR5^+^PD-1^+^ T_FH_ cells (Figure 7B&C) and total number of spleen cells were significantly increased (Figure 7D) which is correlated to enhanced GC number (Figure 7E&F) in CD4-PPARγ^KO^ mice than in control animals. Next, we examined IL-21 and Bcl-6 expression levels, which are important molecules for induction of T_FH_ cells and GC reaction. CD4-PPARγ^KO^ T cells expressed significantly higher level of IL-21 and Bcl-6 (Figure 7G&H) while they have reduced level of Blimp-1 (Figure 7I) in Th2 or Th17 cells suggesting PPARγ negatively regulates Bcl-6 and IL-21 to inhibit T_FH_ cell differentiation and GC reaction. These results suggest that PPARγ inhibits formation of T_FH_ cells and GC reaction via regulation of Bcl-6 and IL-21 to prevent autoimmune disease.

## Discussion

Based on its ligand-dependent functions, PPARγ has been demonstrated to be an important negative regulator in T cells, macrophages, and dendritic cells [9], [24]. Previous studies have also suggested that PPARγ is an important therapeutic target for treating autoimmune disease [5]–[7]. Here, for the first time, we characterized CD4-PPARγ^KO^ mice to investigate the intrinsic role of PPARγ in T cells and autoimmunity related to T_FH_ cells and GC reaction.

We found that, among lymphocytes, PPARγ was expressed predominantly in CD4^+^ T cells. The expression level of PPARγ, however, increased following TCR stimulation. This is consistent with a previous study that reported an increase in PPARγ expression following anti-CD3 antibody stimulation and PPARγ ligand (15d-PGJ2 and ciglitazone) treatment, which it also contributed to the inhibition of T cell proliferation and IL-2 production [9]. Taken together, these results imply that PPARγ expression and TCR stimulation function together to regulate T cell response.

One recent study reported that pioglitazone, a PPARγ ligand, inhibited PPARγ-meditated differentiation of Th17, but not Th1, cells in an autoimmune EAE model, which suggested the possibility of selective regulation of Th17 cells by PPARγ [11]. However, we found significantly increase of all the cytokines, including IFN-γ, IL-13, IL-17, and IL-9 in each T cell differentiation skewing media without affecting differentiation efficiency possibly due to hyper-proliferative property of PPARγ-deficient T cells. This increased T cell sensitivity seems to be correlated with higher AKT and ERK phosphorylation as well as rapid IκBα degradation following TCR stimulation.

Previously PPARγ was studied to inhibit the NF-κB and AP-1 transcription factors in macrophages and T cells [24], [25], [32] and PPARγ agonists were shown to inhibit inflammatory cytokine production in monocytes [33], as well as proliferation or IL-2 production in T cells following TCR stimulation [9]. Our results demonstrated a loss of Sirt1 and Foxo1, two negative regulators of T cell activation, in PPARγ-deficient T cells following TCR stimulation. Sirt1, a histone deacetylase, was shown to inhibit AP-1 and is required for T cell tolerance [26]. Foxo1 is also required for inhibiting T cell activation and acts as a T cell-intrinsic regulator of tolerance [34]. The levels of Sirt1 and Foxo1 expression were significantly down-regulated in PPARγ-deficient T cells following TCR stimulation, suggesting that PPARγ controls the stability or expression of Sirt1 and Foxo1 to inhibit NF-kB or AP-1 activation. In addition, Foxo1 is a positive transcriptional regulator of Sirt1 and is deacetylated by Sirt1 [35]. Analysis of ChIP-seq data from BIOBASE (http://www.biobase-international.com) identified various PPARγ response elements within the Foxo1 promoter region based on the study of genome wide analysis of PPARγ binding region in 3T3-L1 cells [36]. Foxo1 signaling through NF-κB has been shown to mediate proinflammatory cytokine production by interacting together in a synergistic manner in macrophages [37]. Another study also revealed the role of Foxo1 in T cell tolerance by demonstrating that T cell-specific deletion of Foxo1 caused hyper-reactivity of T cells [34]. In addition, Foxo1-deleted CD4 T cells also spontaneously increased T_FH_ cells and GC formation as well as autoantibody production in 8-week-old mice [34]. ChIP analysis examining the interaction of PPARγ on Foxo1 promoter region will be performed in a future study to elucidate more detail mechanism.

Previous studies demonstrated treatment of T cells with a PPARγ agonist increases Foxp3 expression and iTreg generation [10], [38]. However, our results demonstrated that PPARγ is not required for Treg generation suggesting PPARγ agonists possibly increase Foxp3 expression PPARγ independently. One recent study reported that PPARγ was dispensable for Foxp3 expression in Treg cells yet critical for inhibiting colitis [39] while PPARγ was recently shown to influence the migration of Treg cells to visceral adipose tissues by regulating chemokine receptor expression without affecting Treg differentiation [13]. Collectively, these results indicate that PPARγ is required for the *in vivo* suppressive function related to the migration of Treg cells into inflammatory regions, but not Foxp3 expression itself.

This study is the first to characterize CD4-PPARγ^KO^ mice to demonstrate spontaneous autoimmune phenotypes along with T_FH_ cells and GC formation. T_FH_ cells have recently been emerging as a T helper subset expressing Bcl-6 and IL-21 that interacts with B cells during GC formation. CD28- or ICOS-deficient mice exhibit defective GC formation, affinity maturation, and T_FH_ cell development [21], [40]–[43]. CD28 or ICOS costimulatory signals and TCR signals are essential for the induction of T_FH_ cells [40], [41]. Reports have also shown that stronger binding to major histocompatibility complex (MHC) class II and TCR is important for T_FH_ cell development [44]. Therefore, increased AKT phosphorylation and reduced expression of negative regulators to NF-κB in PPARγ-deficient T cells following TCR stimulation could contribute to increased T_FH_ cell generation *in vivo*. We also demonstrate an increased proportion of T_FH_ cells and number of GCs following SRBC immunization in CD4-PPARγ^KO^ mice. In addition, PPARγ deficient Th2 and Th17 cells produced higher IL-21 and Bcl-6 suggesting more T_FH_ cells possibly are generated and contributed GC reaction during antigen stimulation in vivo. One previous study suggested that PPARγ sustains Foxo1 expression [45] and another study demonstrated that mice lacking Foxo1 in T cells exhibited a large population of T_FH_ cells and a decreased number of Treg cells [25], suggesting a molecular mechanism for increase of T_FH_ cell differentiation may involving relationship of PPARγ and Foxo1.

In conclusion, we have revealed the importance of PPARγ in regulating T cell activation related to T_FH_ cells and autoimmunity. Modulating PPARγ to dampen abnormal T cell activation and T_FH_ cell mediated GC reaction can be exploited for treating autoimmune diseases.

## Materials and Methods

### Mice

B6.129-Pparg^tm2Rev^/J (PPARγ^fl/fl^) mice were obtained from the Jackson Laboratory (Bar Harbor, ME). To generate CD4-specific PPARγ knockout mice, PPARγ^fl/fl^ mice were crossed with CD4-Cre^+/−^ transgenic mice. All mice were maintained at the Yale University and Hanyang University mouse facilities under pathogen-free conditions.

### Ethics statement

All animal protocols used in this study were approved by the Yale Institutional Animal Care and Use Committee and Hanyang Animal Care and Use Committee.

### Western blot analysis

CD4^+^ T cells were enriched from isolated splenocytes using antibody-coated magnetic beads. The negatively selected CD4^+^ T cells were labeled with CD62L MACS bead (Miltenyi Biotec, Bergisch Gladbach, Germany) to purify CD4^+^CD62L^+^ naïve T cells. The purity of naïve T cells was 95∼96% (data not shown). The isolated naïve T cells were stimulated with anti-CD3 and anti-CD28 antibody coated Dynabeads (Life Technologies, Carlsbad, CA) at 37°C for 0, 5, 10, and 20 min. The cells were then lysed with RIPA buffer (Cell Signaling Technology, Danvers, MA) on ice for 20 min and the protein content was determined using the Pierce BCA protein assay kit (Thermo Fisher Scientific, Waltham, MA). The proteins were resolved on 9% SDS polyacrylamide gels and transferred onto PVDF membrane (Millipore, Temecula, CA). After blocking with 5% skim milk, the membrane was incubated with specific antibodies against P-ERK, P-AKT, P-P65, IκBα, Foxo1, Sirt1 and β-actin (Cell Signaling Technology) overnight at 4°C. HRP-conjugated secondary antibody followed by incubation with Supersignal West Femto ECL solution (Thermo Fisher scientific) were used to detect primary antibody binding.

### Flow cytometry

Splenocytes and mesenteric lymph node cells were isolated and then stained with anti-mouse CD4-APC, CD8-PerCP-Cy5.5, CD19-PE, CD44-PE, CD62L-FITC, CD69-FITC, IL-7Rα-PE, CD138-APC, PSGL-1-APC, CXCR5-PE, PD-1-FITC, GL-7-FITC, CD95-PE-Cy7, B220-Alexa647 and NK1.1-FITC (eBioscience, San Diego, CA) antibodies for 15 min at 4°C. To assess intracellular cytokine levels, cells were differentiated for 5 days and then re-stimulated with Cell Stimulation Cocktail plus Protein Transport Inhibitors reagent (eBioscience) for 5 h. Then, the cells were fixed, permeabilized, and stained with anti-mouse IFN-γ-FITC, IL-4-PE, IL-17-APC, and IL-9-APC antibodies. Intracellular Foxp3 staining was performed using the Foxp3 Staining Kit (eBioscience). To determine the purity of isolated CD4^+^CD25^−^ T cells, these cells were stained with anti-CD4 and anti-CD25, and followed by FACS analysis.

### Quantitative real-time PCR

RNA from FACS-sorted CD4^+^, CD8^+^, and CD19^+^ cells was isolated using the RNeasy Mini kit (Qiagen, Venlo, Limburg) according to the manufacturer's protocol. cDNA synthesis was performed using the iScript kit (Bio-Rad, Hercules, CA). Real-time PCR was performed on a Bio-Rad CFX Connect real-time PCR detection system using iQ SYBR Green Supermix (Bio-Rad). The level of PPARγ expression was assessed in triplicate with specific primers and normalized to β-actin. The primer sequences used were: PPARγ (forward): 5′-CCAGAGTCTGCTGATCTGCG-3′; PPARγ (reverse): 5′-GCCACCTCTTTGCTCTGCTC-3′; Bcl-6 (forward): 5′-CCGGCACGCTAGTGATGTT-3′; Bcl-6 (reverse): 5′-TGTCTTATGGGCTCTAAACTGCT-3′; IL-21 (forward): 5′-GGACCCTTGTCTGTCTGGTAG-3′; IL-21 (reverse): 5′-TGTGGAGCTGATAGAAGTTCAGG-3′; Blimp-1 (forward): 5′-TTCTTGTGTGGTATTGTCGGGACTT-3′; Blimp-1 (reverse): 5′-TTGGGGACACTCTTTGGGTAGAGTT-3′; β-actin (forward): 5′-TGTCCCTGTATGCCTCTGGT-3′; and β-actin (reverse): 5′-CACGCACGATTTCCCTCTC-3′ (34).

### ELISA

Cytokine levels in the supernatants of polarized Th1, Th2, Th17, and Th9 cultures were determined by ELISA using antibodies against mouse IL-2 (Biolegend, San Diego, CA), IFN-γ, IL-13, IL-17 and IL-9 (eBioscience) according to the manufacturer's instructions. Anti-dsDNA antibody in mouse serum was determined by ELISA (Alpha Diagnostic International Inc, San Antonio, TX).

### Confocal microscopy

Mice aged 6 to 8 weeks old were immunized with sheep red blood cells (SRBC) and spleens were harvested 9 days later. Spleens from 1-year-old mice were also collected to analyze T_FH_ cells and GC formation. The tissues were frozen in OCT reagent and sectioned into 7-µm slices. The frozen sections were stained with anti-PNA-FITC (Sigma-Aldrich, St. Louis, MO), IgD-PE, and CD4-APC (eBioscience) overnight at 4°C. After washing, Anti-Fade reagent (Invitrogen, Life Technologies, Carlsbad, CA) was added to the slides, which were visualized using a Leica DM IRE2 confocal microscope.

### T cell activation, proliferation, and differentiation

Spleens were isolated from mice and a single-cell suspension was prepared. The single cell suspension was stained against CD4 and CD25, and CD4^+^CD25^−^ T cells were purified by MoFlo (Beckman Coulter, Inc., Brea, CA), while naïve CD4 T cells were isolated using the CD4^+^CD62L^+^ T cell isolation kit (Miltenyi Biotec, Bergisch Gladbach, Germany) according to the manufacturer's instructions. Purified T cells were stimulated with plate-bound anti-CD3 and soluble anti-CD28 antibodies in 96-well plates and differentiated under the following subset-specific conditions: IL-12 (2 ng/ml), IL-2 (50 U/ml), and anti-IL-4 (5 µg/ml) for Th1 differentiation; IL-4 (30 ng/ml), IL-2 (50 U/ml), and anti-IFN-γ (5 µg/ml) for Th2 differentiation; TGF-β (2 ng/ml), IL-4 (30 ng/ml), and anti-IFN-γ (5 µg/ml) for Th9 differentiation; TGF-β (0.5 ng/ml), IL-6 (30 ng/ml), IL-23 (20 ng/ml), IL-1β (20 ng/ml), anti-IFN-γ (5 µg/ml), and anti-IL-4 (5 µg/ml) for Th17 differentiation; TGF-β (5 ng/ml) and IL-2 (100 U/ml) for Treg differentiation. The cells were pulsed with H^3^-thymidine (1 µCi/well) overnight and H^3^-thymidine incorporation was measured to assess T cell proliferation.

### Histology

Kidney tissues from 1-year-old mice were isolated, fixed, embedded in paraffin, and stained with hematoxylin and eosin. For each mouse, the level of kidney inflammation and more than 15 glomerular, tubular, or interstitial areas were evaluated and scored blindly for glomerular cellularity, leukocyte infiltration, severity of tubular lesions, mesangial matrix expansion, crescent formation, and interstitial mononuclear cell infiltration in the medulla and cortex. The severity of kidney lesions was determined by scoring each feature (from 0 to 3) and then calculating the mean of each set of scores. For example, glomerular inflammation was scored as follows: 0 =  normal or no inflammatory cells; 1 =  few inflammatory cells; 2 =  moderate inflammation; and 3 =  severe lymphocyte infiltration.

### Statistical analysis

All data were analyzed statistically analyzed with the Student's t-test and Mann-Whitney test using Prism5 (GraphPad, San Diego, CA). p-values (P) less than 0.05 were considered statistically significant.

## Supporting Information

Figure S1 **Characterization of lymphocyte populations in CD4-PPARγ^KO^ mice.** (A-D) Thymic and (E-H) splenic CD4^+^, CD8^+^, and Foxp3^+^ populations from 6- to 8-week-old female wild type and CD4-PPARγ^KO^ mice were analyzed by flow cytometry. Total CD4^+^ or CD8^+^ cells were gated from live cells while Foxp3^+^ cells were gated from CD4^+^ T cells. (I, J) Splenic B and NK cells, which were gated from live cells, and (K, L) CD62L and CD44 populations gated from CD4^+^ T cells of 6- to 8- week-old female littermate control (Cre-) and CD4-PPARγ^KO^ (Cre+) mice were analyzed by flow cytometry. Values represent the mean ± SEM. *P<0.05.
(DOCX)

## References

[1] Chawla A, Repa JJ, Evans RM, Mangelsdorf DJ (2001) Nuclear receptors and lipid physiology: opening the X-files. Science 294: 1866–1870. 11729302 10.1126/science.294.5548.1866

[2] Evans RM (1988) The steroid and thyroid hormone receptor superfamily. Science 240: 889–895. 3283939 10.1126/science.3283939 PMC6159881

[3] Glass CK, Ogawa S (2006) Combinatorial roles of nuclear receptors in inflammation and immunity. Nat Rev Immunol 6: 44–55. 16493426 10.1038/nri1748

[4] Choi JM, Bothwell AL (2012) The nuclear receptor PPARs as important regulators of T-cell functions and autoimmune diseases. Mol Cells 33: 217–222. 22382683 10.1007/s10059-012-2297-y PMC3887706

[5] Lehmann JM, Moore LB, Smith-Oliver TA, Wilkison WO, Willson TM, et al (1995) An antidiabetic thiazolidinedione is a high affinity ligand for peroxisome proliferator-activated receptor gamma (PPAR gamma). J Biol Chem 270: 12953–12956. 7768881 10.1074/jbc.270.22.12953

[6] Hsueh WA, Law RE (2001) PPARgamma and atherosclerosis: effects on cell growth and movement. Arterioscler Thromb Vasc Biol 21: 1891–1895. 11742860 10.1161/hq1201.100261

[7] Desreumaux P, Dubuquoy L, Nutten S, Peuchmaur M, Englaro W, et al (2001) Attenuation of colon inflammation through activators of the retinoid X receptor (RXR)/peroxisome proliferator-activated receptor gamma (PPARgamma) heterodimer. A basis for new therapeutic strategies. J Exp Med 193: 827–838. 11283155 10.1084/jem.193.7.827 PMC2193371

[8] Gervois P, Fruchart JC, Staels B (2007) Drug Insight: mechanisms of action and therapeutic applications for agonists of peroxisome proliferator-activated receptors. Nat Clin Pract Endocrinol Metab 3: 145–156. 17237841 10.1038/ncpendmet0397

[9] Clark RB, Bishop-Bailey D, Estrada-Hernandez T, Hla T, Puddington L, et al (2000) The nuclear receptor PPAR gamma and immunoregulation: PPAR gamma mediates inhibition of helper T cell responses. J Immunol 164: 1364–1371. 10640751 10.4049/jimmunol.164.3.1364

[10] Wohlfert EA, Nichols FC, Nevius E, Clark RB (2007) Peroxisome proliferator-activated receptor gamma (PPARgamma) and immunoregulation: enhancement of regulatory T cells through PPARgamma-dependent and -independent mechanisms. J Immunol 178: 4129–4135. 17371968 10.4049/jimmunol.178.7.4129

[11] Klotz L, Burgdorf S, Dani I, Saijo K, Flossdorf J, et al (2009) The nuclear receptor PPAR gamma selectively inhibits Th17 differentiation in a T cell-intrinsic fashion and suppresses CNS autoimmunity. J Exp Med 206: 2079–2089. 19737866 10.1084/jem.20082771 PMC2757877

[12] Tobiasova Z, Zhang L, Yi T, Qin L, Manes TD, et al (2011) Peroxisome proliferator-activated receptor-gamma agonists prevent in vivo remodeling of human artery induced by alloreactive T cells. Circulation 124: 196–205. 21690493 10.1161/CIRCULATIONAHA.110.015396 PMC3347886

[13] Cipolletta D, Feuerer M, Li A, Kamei N, Lee J, et al (2012) PPAR-gamma is a major driver of the accumulation and phenotype of adipose tissue Treg cells. Nature 486: 549–553. 22722857 10.1038/nature11132 PMC3387339

[14] King C, Tangye SG, Mackay CR (2008) T follicular helper (TFH) cells in normal and dysregulated immune responses. Annu Rev Immunol 26: 741–766. 18173374 10.1146/annurev.immunol.26.021607.090344

[15] Craft JE (2012) Follicular helper T cells in immunity and systemic autoimmunity. Nat Rev Rheumatol 8: 337–347. 22549246 10.1038/nrrheum.2012.58 PMC3604997

[16] Sweet RA, Lee SK, Vinuesa CG (2012) Developing connections amongst key cytokines and dysregulated germinal centers in autoimmunity. Curr Opin Immunol 24: 658–664. 23123277 10.1016/j.coi.2012.10.003

[17] Crotty S (2011) Follicular helper CD4 T cells (TFH). Annu Rev Immunol 29: 621–663. 21314428 10.1146/annurev-immunol-031210-101400

[18] Breitfeld D, Ohl L, Kremmer E, Ellwart J, Sallusto F, et al (2000) Follicular B helper T cells express CXC chemokine receptor 5, localize to B cell follicles, and support immunoglobulin production. J Exp Med 192: 1545–1552. 11104797 10.1084/jem.192.11.1545 PMC2193094

[19] Schaerli P, Willimann K, Lang AB, Lipp M, Loetscher P, et al (2000) CXC chemokine receptor 5 expression defines follicular homing T cells with B cell helper function. J Exp Med 192: 1553–1562. 11104798 10.1084/jem.192.11.1553 PMC2193097

[20] Nurieva RI, Chung Y, Martinez GJ, Yang XO, Tanaka S, et al (2009) Bcl6 mediates the development of T follicular helper cells. Science 325: 1001–1005. 19628815 10.1126/science.1176676 PMC2857334

[21] Choi YS, Kageyama R, Eto D, Escobar TC, Johnston RJ, et al (2011) ICOS receptor instructs T follicular helper cell versus effector cell differentiation via induction of the transcriptional repressor Bcl6. Immunity 34: 932–946. 21636296 10.1016/j.immuni.2011.03.023 PMC3124577

[22] Simpson N, Gatenby PA, Wilson A, Malik S, Fulcher DA, et al (2010) Expansion of circulating T cells resembling follicular helper T cells is a fixed phenotype that identifies a subset of severe systemic lupus erythematosus. Arthritis Rheum 62: 234–244. 20039395 10.1002/art.25032

[23] Park HJ, Kim DH, Lim SH, Kim WJ, Youn J, et al (2014) Insights into the Role of Follicular Helper T Cells in Autoimmunity. Immune network 14: 21–29. 24605077 10.4110/in.2014.14.1.21 PMC3942504

[24] Ricote M, Li AC, Willson TM, Kelly CJ, Glass CK (1998) The peroxisome proliferator-activated receptor-gamma is a negative regulator of macrophage activation. Nature 391: 79–82. 9422508 10.1038/34178

[25] Kerdiles YM, Stone EL, Beisner DR, McGargill MA, Ch'en IL, et al (2010) Foxo transcription factors control regulatory T cell development and function. Immunity 33: 890–904. 21167754 10.1016/j.immuni.2010.12.002 PMC3034255

[26] Zhang J, Lee SM, Shannon S, Gao B, Chen W, et al (2009) The type III histone deacetylase Sirt1 is essential for maintenance of T cell tolerance in mice. J Clin Invest 119: 3048–3058. 19729833 10.1172/JCI38902 PMC2752073

[27] Xue HH, Kovanen PE, Pise-Masison CA, Berg M, Radovich MF, et al (2002) IL-2 negatively regulates IL-7 receptor alpha chain expression in activated T lymphocytes. Proc Natl Acad Sci U S A 99: 13759–13764. 12354940 10.1073/pnas.212214999 PMC129770

[28] Liu YJ, Malisan F, de Bouteiller O, Guret C, Lebecque S, et al (1996) Within germinal centers, isotype switching of immunoglobulin genes occurs after the onset of somatic mutation. Immunity 4: 241–250. 8624814 10.1016/s1074-7613(00)80432-x

[29] MacLennan IC (1994) Germinal centers. Annu Rev Immunol 12: 117–139. 8011279 10.1146/annurev.iy.12.040194.001001

[30] Berek C, Berger A, Apel M (1991) Maturation of the immune response in germinal centers. Cell 67: 1121–1129. 1760840 10.1016/0092-8674(91)90289-b

[31] Poholek AC, Hansen K, Hernandez SG, Eto D, Chandele A, et al (2010) In vivo regulation of Bcl6 and T follicular helper cell development. J Immunol 185: 313–326. 20519643 10.4049/jimmunol.0904023 PMC2891136

[32] Yang XY, Wang LH, Chen T, Hodge DR, Resau JH, et al (2000) Activation of human T lymphocytes is inhibited by peroxisome proliferator-activated receptor gamma (PPARgamma) agonists. PPARgamma co-association with transcription factor NFAT. J Biol Chem 275: 4541–4544. 10671476 10.1074/jbc.275.7.4541

[33] Jiang C, Ting AT, Seed B (1998) PPAR-gamma agonists inhibit production of monocyte inflammatory cytokines. Nature 391: 82–86. 9422509 10.1038/34184

[34] Ouyang W, Beckett O, Flavell RA, Li MO (2009) An essential role of the Forkhead-box transcription factor Foxo1 in control of T cell homeostasis and tolerance. Immunity 30: 358–371. 19285438 10.1016/j.immuni.2009.02.003 PMC2692529

[35] Xiong S, Salazar G, Patrushev N, Alexander RW (2011) FoxO1 mediates an autofeedback loop regulating SIRT1 expression. J Biol Chem 286: 5289–5299. 21149440 10.1074/jbc.M110.163667 PMC3037641

[36] Lefterova MI, Zhang Y, Steger DJ, Schupp M, Schug J, et al (2008) PPARgamma and C/EBP factors orchestrate adipocyte biology via adjacent binding on a genome-wide scale. Genes Dev 22: 2941–2952. 18981473 10.1101/gad.1709008 PMC2577797

[37] Su D, Coudriet GM, Hyun Kim D, Lu Y, Perdomo G, et al (2009) FoxO1 links insulin resistance to proinflammatory cytokine IL-1beta production in macrophages. Diabetes 58: 2624–2633. 19651810 10.2337/db09-0232 PMC2768186

[38] Baratelli F, Lin Y, Zhu L, Yang SC, Heuze-Vourc'h N, et al (2005) Prostaglandin E2 induces FOXP3 gene expression and T regulatory cell function in human CD4+ T cells. J Immunol 175: 1483–1490. 16034085 10.4049/jimmunol.175.3.1483

[39] Hontecillas R, Bassaganya-Riera J (2007) Peroxisome proliferator-activated receptor gamma is required for regulatory CD4+ T cell-mediated protection against colitis. J Immunol 178: 2940–2949. 17312139 10.4049/jimmunol.178.5.2940

[40] Vogelzang A, McGuire HM, Yu D, Sprent J, Mackay CR, et al (2008) A fundamental role for interleukin-21 in the generation of T follicular helper cells. Immunity 29: 127–137. 18602282 10.1016/j.immuni.2008.06.001

[41] Walker LS, Gulbranson-Judge A, Flynn S, Brocker T, Raykundalia C, et al (1999) Compromised OX40 function in CD28-deficient mice is linked with failure to develop CXC chemokine receptor 5-positive CD4 cells and germinal centers. J Exp Med 190: 1115–1122. 10523609 10.1084/jem.190.8.1115 PMC2195670

[42] Akiba H, Takeda K, Kojima Y, Usui Y, Harada N, et al (2005) The role of ICOS in the CXCR5+ follicular B helper T cell maintenance in vivo. J Immunol 175: 2340–2348. 16081804 10.4049/jimmunol.175.4.2340

[43] Lane P, Burdet C, Hubele S, Scheidegger D, Muller U, et al (1994) B cell function in mice transgenic for mCTLA4-H gamma 1: lack of germinal centers correlated with poor affinity maturation and class switching despite normal priming of CD4+ T cells. J Exp Med 179: 819–830. 7509361 10.1084/jem.179.3.819 PMC2191407

[44] Fazilleau N, McHeyzer-Williams LJ, Rosen H, McHeyzer-Williams MG (2009) The function of follicular helper T cells is regulated by the strength of T cell antigen receptor binding. Nat Immunol 10: 375–384. 19252493 10.1038/ni.1704 PMC2712297

[45] Dowell P, Otto TC, Adi S, Lane MD (2003) Convergence of peroxisome proliferator-activated receptor gamma and Foxo1 signaling pathways. J Biol Chem 278: 45485–45491. 12966085 10.1074/jbc.M309069200

