# Supplementary material for: PPARγ Negatively Regulates T Cell Activation to Prevent Follicular Helper T Cells and Germinal Center Formation
Source: PLoS One. 2014 Jun 12;9(6):e99127. doi: 10.1371/journal.pone.0099127 (PMC4055678; doi:10.1371/journal.pone.0099127)
Supplement: Figure S1 — Characterization of lymphocyte populations in CD4-PPARγKO mice. (A-D) Thymic and (E-H) splenic CD4+, CD8+, and Foxp3+ populations from 6- to 8-week-old female wild type and CD4-PPARγKO mice were analyzed by flow cytometry. Total CD4+ or CD8+ cells were gated from live cells while Foxp3+ cells were gated from CD4+ T cells. (I, J) Splenic B and NK cells, which were gated from live cells, and (K, L) CD62L and CD44 populations gated from CD4+ T cells of 6- to 8- week-old female littermate control (Cre-) and CD4-PPARγKO (Cre+) mice were analyzed by flow cytometry. Values represent the mean ± SEM. *P<0.05. (DOCX) [file pone.0099127.s001.docx]

**
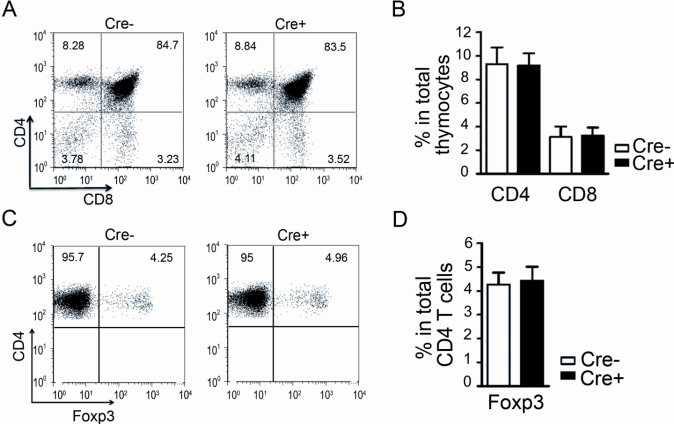
**

**
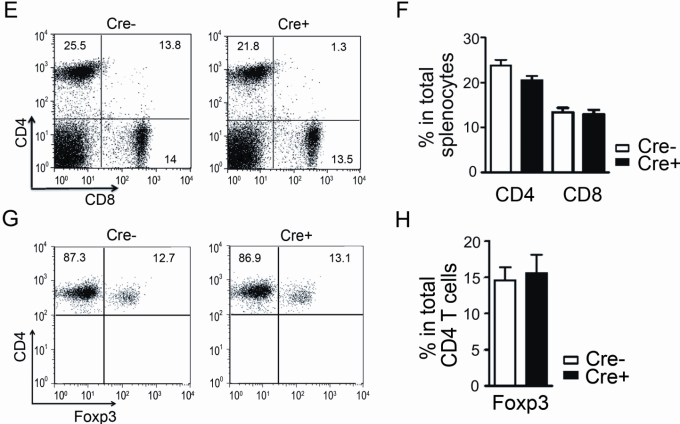
**


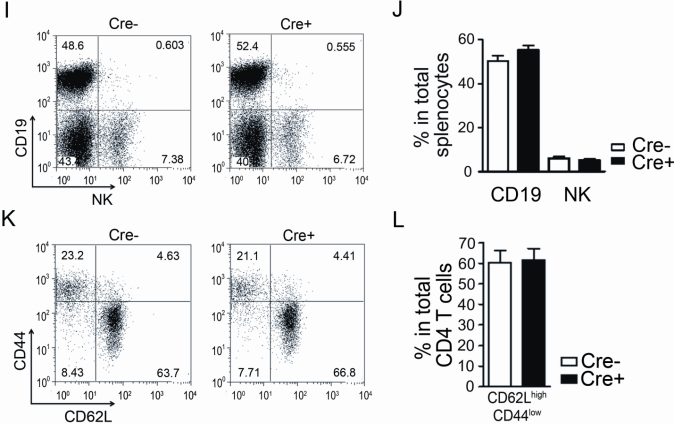


**Figure S1 Characterization of lymphocyte populations in CD4-PPARγ^KO^ mice.** (A-D) Thymic and (E-H) splenic CD4^+^, CD8^+^, and Foxp3^+^ populations from 6- to 8-week-old female wild type and CD4-PPARγ^KO^ mice were analyzed by flow cytometry. Total CD4^+^ or CD8^+^ cells were gated from live cells while Foxp3^+^ cells were gated from CD4^+^ T cells. (I, J) Splenic B and NK cells, which were gated from live cells, and (K, L) CD62L and CD44 populations gated from CD4^+^ T cells of 6- to 8- week-old female littermate control (Cre-) and CD4-PPARγ^KO^ (Cre+) mice were analyzed by flow cytometry. Values represent the mean ± SEM. **P* < 0.05.
